# Fruit Pulps Enhance Longevity and Parasitism of *Trichopria drosophilae*, a Pupal Parasitoid of Drosophilid Flies

**DOI:** 10.3390/insects17090976

**Published:** 2026-09-21

**Authors:** Fan Yang, Zehua Wang, Qiufei Li, Ren Li, Guanghang Qiao, Ziwei Zhu, Shanning Wang

**Affiliations:** Institute of Plant Protection, Beijing Academy of Agriculture and Forestry Sciences/Key Laboratory of Environment Friendly Management on Fruit and Vegetable Pests in North China (Co-Construction by Ministry and Province), Ministry of Agriculture and Rural Affairs, Beijing 100093, China; yangfan@baafs.net.cn (F.Y.); wangzehua200707@163.com (Z.W.); 18897755317@163.com (Q.L.); liren@baafs.net.cn (R.L.); qghang98@126.com (G.Q.); zhuziwei@baafs.net.cn (Z.Z.)

**Keywords:** *Trichopria drosophilae*, fruit resources, fitness, parasitoid performance, biological control

## Abstract

*Trichopria drosophilae* is a cosmopolitan pupal parasitoid of drosophilid pests, including the globally destructive *Drosophila suzukii*. Improving parasitoid fitness through nutritional supplementation is a promising strategy for biological control, yet the suitability of field-available food resources remains largely unexplored. Here, we assessed how adult diet influences longevity, survival, parasitism, offspring production, and sex ratio of *T. drosophilae*. Seven dietary treatments were tested: water control, 10% honey solution, banana pulp, blueberry pulp, cherry pulp, *Orychophragmus violaceus* flowers alone, and flowers plus water. Fruit pulps, especially cherry, dramatically prolonged adult longevity to over 28 days, matching or surpassing the honey standard. Wasps fed *O. violaceus* flowers alone survived only approximately five days; however, providing water together with flowers restored survival to a level comparable to the water control, indicating that water deprivation was an important (at least partial) cause of this short lifespan, and no evidence was found that *O. violaceus* flowers provide accessible nectar. In a density-dependent parasitism assay across six host densities (5–30 pupae), a significant diet-by-density interaction was detected: parasitism rates were low at low host densities and increased with host density, whereas at intermediate and high densities (15–30 hosts), fruit-fed wasps parasitized significantly more hosts than the water control (blueberry at 15 hosts; banana at 20 hosts; all three fruit treatments at 25 and 30 hosts). Across all densities, fruit-fed wasps parasitized significantly more hosts overall than those receiving water or honey alone. Consistently, total offspring and female emergence were greater in fruit-fed groups than in water-fed wasps. Offspring sex ratio was unaffected by diet. These results demonstrate that fruit pulps are highly effective nutritional resources that enhance both survival and reproductive success of *T. drosophilae* in a density-dependent manner, whereas *O. violaceus* flowers alone are insufficient to sustain parasitoid survival. Our findings highlight the potential of fruit resources as natural food subsidies for parasitoids and underscore the need to consider floral accessibility when selecting plants for conservation biological control.

## Simple Summary

*Trichopria drosophilae* is a pupal parasitoid of drosophilid flies, including the invasive pest *Drosophila suzukii*. The success of biological control using this parasitoid depends on the availability of suitable food resources for adults. We tested how common field-available foods—water, honey solution, banana, blueberry and cherry pulps, and flowers of the cover crop *Orychophragmus violaceus*—affect the survival and parasitic performance of *T. drosophilae*. Fruit pulps, especially cherry, dramatically extended adult lifespan to over 28 days, comparable to or exceeding a honey diet, and substantially increased parasitism rates, particularly when host densities were high. Wasps that received only *O. violaceus* flowers lived about five days; however, adding water together with flowers restored survival to control levels, indicating that water deprivation was an important contributor to this short lifespan, whereas the flowers provided no accessible nectar. Offspring sex ratio was not affected by diet. Our findings suggest that fruit pulps can serve as effective nutritional resources for *T. drosophilae*, while the accessibility of floral resources should be considered when selecting plants for conservation biological control.

## 1. Introduction

*Trichopria drosophilae* (Hymenoptera: Diapriidae) is an important pupal endoparasitoid of the globally significant pest *Drosophila suzukii* (Spotted Wing Drosophila) and other drosophilid species [1]. As a key native natural enemy, its potential for augmentative biological control in susceptible crops, such as soft-skinned fruits, is increasingly recognized [2,3,4], with evidence suggesting that augmentative releases can suppress pest populations in unmanaged areas surrounding crops, thereby mitigating outbreak severity in orchards [5]. However, the success of parasitoids in biological control programs is critically dependent on their fitness and persistence in the target environment [6].

Adult nutrition is a pivotal factor influencing key fitness parameters in parasitoids, including longevity, fecundity, and host-searching capacity. In nature, many parasitoid species rely on carbohydrate-rich food sources, such as floral nectar and honeydew, to fuel their activities and extend lifespan [7,8]. For *T. drosophilae*, access to sugar resources like a 10% honey solution has been shown to be optimal for maintaining adult longevity in laboratory settings [9]. While such artificial diets are standard in laboratory rearing [1,10], their availability in complex agricultural landscapes is limited. Therefore, identifying effective, field-available food resources is essential for enhancing parasitoid establishment and efficacy [11].

Conservation biological control commonly employs flowering plants to provide nutritional support for parasitoids [12,13,14], with species like buckwheat (*Fagopyrum esculentum*) significantly enhancing the lifespan and fecundity of *T. drosophilae* [15]. However, the benefits of floral resources are highly species-specific and may even be detrimental to some parasitoids [16,17], while the nutritional value of sugars varies, with monosaccharides and disaccharides generally being more effective [18]. Notably, *T. drosophilae*—a species adapted to cooler early-spring conditions [19,20] and often dominant in early-season field collections [21]—emerges as an adult during a period that overlaps with the flowering window of early-blooming plants such as *Orychophragmus violaceus* (flowering from early March for about 40 days [22]). *O. violaceus* is also among the most common and abundant early-spring plants in northern Chinese orchards and urban green spaces, where it is frequently retained as a green-manure ground cover [23,24]. This phenological synchrony suggests that early-season floral resources like *O. violaceus* could serve as potential nectar sources to support *T. drosophilae* in the field, though their actual suitability remains to be evaluated.

Beyond floral resources, host-associated environments present alternative nutritional opportunities. *D. suzukii* develops within fruit, and resources like fruit juices or pulp from damaged or overripe fruit are abundant in infested crops [11]. These resources may represent a co-evolved or readily exploitable food source for associated parasitoids. Indeed, the fruit species in which *D. suzukii* pupates can influence the development and performance of the parasitoid *T. drosophilae* [25]. However, the relative value of these host-associated fruit resources as adult food, compared to standard artificial diets or floral provisions, remains largely unexplored for *T. drosophilae*.

This study aims to comprehensively evaluate the effects of various field-relevant food resources on the fitness of *T. drosophilae*. We compared a conventional artificial diet (10% honey solution) with novel resources, including the flowers of a common orchard green manure plant, *O. violaceus* [26,27], and the pulp of fruits commonly infested by drosophilids (banana, blueberry, and cherry). Parasitism and offspring production were quantified using pupae of *Drosophila melanogaster*, a factitious host routinely used for this parasitoid [28,29], which allowed synchronized, age-standardized host production. We hypothesized that these fruit-based resources would serve as excellent nutritional supplements. Specifically, we assessed (1) adult longevity and survival under different diets, (2) parasitism rate and reproductive output (wasp emergence) when provided with these foods, and (3) potential effects on offspring sex ratio. The findings are expected to provide practical insights for formulating conservation biological control strategies to support *T. drosophilae* populations in agricultural systems.

## 2. Materials and Methods

### 2.1. Insect Collection and Rearing

The *D. melanogaster* colony in this study is a long-term laboratory population (no specific strain designation), maintained since 2020. *D. melanogaster* was chosen as a factitious host because it can be mass-reared year-round with highly synchronized pupation, providing age-standardized pupae, and it is an established host on which *T. drosophilae* develops readily [28,29]. The flies were reared on an artificial diet prepared with the following ingredients: 250 g wheat bran, 130 g sucrose (Sinopharm Chemical Reagent Co., Ltd., Shanghai, China), 35 g brewer’s yeast powder, 1 g methylparaben (VWR chemicals, Solon, OH, USA), Vanderzant vitamin mix for insects (Sigma-Aldrich, St. Louis, MO, USA), and 600 mL double-distilled H_2_O. The prepared diet was dispensed into small containers (upper diameter: 12 cm, bottom diameter: 9.3 cm, height: 7.5 cm, volume: 450 mL). These containers, left uncovered, were placed inside rearing cages (35 × 35 × 35 cm, 120 mesh) containing adult *D. melanogaster* for oviposition. After 48 h, the containers were removed, covered with a mesh lid, and maintained for larval development and pupation. Newly formed pupae, recognizable by their pale colour, were collected within 24 h of puparium formation for use in the parasitism assay.

Adult parasitoid wasps, *T. drosophilae*, were originally collected in 2022 from the cherry experimental base at the Institute of Forestry and Pomology, Beijing Academy of Agriculture and Forestry Sciences (39°41′ N, 116°42′ E). Species identity was confirmed both morphologically and molecularly. Morphological identification followed the diagnostic characters of Gumovsky et al. [30], principally the antennal structure (distinctly 3-segmented club in females; elongate flagellar segments with whorls of long hairs in males), the keel-less mesoscutellum, and the proportions of the petiole and gaster. For molecular confirmation, a *COI* fragment was amplified with the universal primers LCO1490/HCO2198 [31] and the sequence was deposited in GenBank under accession number PZ949255. The parasitoid colony was maintained on pupae of the laboratory-reared *D. melanogaster.* Adult wasps were housed in similar rearing cages (35 × 35 × 35 cm, 120 mesh) and provided with a 10% honey-water solution. The laboratory colony remains in culture, and parasitoid specimens are available from the authors upon request. To obtain parasitoids of a known age, containers holding *D. melanogaster* pupae (identical to those described above) were introduced into cages containing adult wasps for parasitization. After a 24-h exposure period, the wasps were removed. Experimental individuals were selected as active wasps of relatively large body size.

All insects were reared in a walk-in climate-controlled room maintained at 24 ± 2 °C, 50 ± 5% relative humidity, and under a photoperiod of 14:10 h (L:D).

### 2.2. Parasitoid Adult Longevity and Survival Assay

The effects of different food sources on adult parasitoid longevity and survival were assessed. The assay was conducted using the small rearing containers (upper diameter: 12 cm; lower diameter: 9.3 cm; height: 7.5 cm; volume: 450 mL) covered with a mesh lid. Each container was provisioned with a single, newly emerged pair of adult wasps (one female and one male, paired on the day of emergence and kept together throughout the assay). The wasps had no prior contact with host pupae and were therefore host-naïve. These newly emerged wasps had received no food before the experiment; each assigned diet was provided from the day of emergence and replenished daily until death.

The wasps were assigned to one of seven dietary treatments: (1) Water Control, (2) 10% Honey solution, (3) Water + Flower, (4) Flower only, (5) Banana, (6) Blueberry, and (7) Cherry. The flower provided in the relevant treatments was from *Orychophragmus violaceus*, a common early-spring green manure plant in northern Chinese orchards. *O. violaceus* plants were originally collected from the cherry experimental base and subsequently maintained in pots in a glasshouse; fresh flowers were picked daily and replaced to ensure consistent quality and availability. Water and the 10% honey solution were provided in 1.5 mL microcentrifuge tubes plugged with absorbent cotton. Fresh fruit flesh (hereafter referred to as “fruit pulp”) was offered as pieces: banana was presented as a 1.0 cm thick cube, and individual blueberries and cherries were cut in half, with the cut surfaces exposed and accessible to the wasps. In the fruit treatments, fruit pulp was the only food source provided. All fruits were ripe, and all food sources were replaced daily to minimize fermentation. The fruits were purchased from online retailers: blueberries were produced in Yunnan Province (China), cherries were sweet cherries of the ‘Tieton’ cultivar (locally known as ‘Meizao’) from Dalian, Liaoning Province (China), and bananas were imported from Ecuador.

The number of replicates (each consisting of one pair in one container) was 30 for the 10% Honey solution treatment and 15 for each of the other six treatments. During the experiment, any parasitoids that escaped or were otherwise lost from their containers were recorded, and their corresponding data points were excluded from the final analysis (marked as NA). The survival of wasps in each container was checked and recorded daily until all individuals had died. The assay was performed under the same controlled environmental conditions as described for insect rearing (Section 2.1).

### 2.3. Parasitism and Offspring Production Assay

To evaluate the effects of adult diet on parasitism success and subsequent offspring production, age-standardized host pupae of *D. melanogaster* (newly formed, pale-coloured pupae collected within 24 h of puparium formation) were offered to parasitoid pairs at six different densities (5, 10, 15, 20, 25, and 30 pupae per replicate), chosen to span a gradient from host-limiting to non-limiting conditions. Each density was replicated six times, giving 36 replicate pairs and 630 host pupae per diet treatment (33 pairs for Cherry after excluding three lost replicates); host provisioning (5–30 pupae per 24-h replicate) follows published protocols for this parasitoid [32]. For each replicate, a single mated pair (5–7 days old) was introduced into a rearing bowl containing the host pupae placed in a 3.5 cm diameter Petri dish. Females and males had been held together continuously since adult emergence to ensure mating; all wasps were host-naïve, because no host pupae were provided before the assay. Before the assay, these 5–7-day-old pairs had been maintained on 10% honey solution (the standard colony diet) throughout the mating period. Based on the longevity assay, the *O. violaceus* treatment was not included in this experiment due to its poor performance. The parasitoids were provided with one of the five diet treatments as described in the longevity assay, and allowed to forage and oviposit for 24 h, after which they were removed. Exposed pupae were incubated under standard rearing conditions until fly or parasitoid emergence.

All intact, uneclosed pupae were individually subjected to DNA extraction and species-specific PCR to determine parasitism status, allowing identification of parasitized hosts irrespective of parasitoid emergence. DNA was extracted by homogenizing each pupa in 40 μL of QuickExtract™ DNA Extraction Solution 1.0 (LGC Science (Shanghai) Ltd., Shanghai, China) with a 1.5-mm steel bead in a Tissuelyser-192L (Shanghai Jingxin Industrial Development Co., Ltd., Shanghai, China) at 60 Hz for 100 s, followed by incubation at 65 °C for 6 min and 98 °C for 90 s. Each 20-μL PCR contained 10.0 μL of 2× Multiplex PCR Buffer, 0.1 μL of Multiplex PCR Enzyme Mix (TaKaRa, Kusatsu, Shiga, Japan; RR062A), 2.0 μL of each primer (10 μM), and 1.0 μL of DNA template, made up with ddH_2_O. Cycling conditions were 94 °C for 1 min; 35 cycles of 94 °C for 30 s, 56 °C for 30 s, and 72 °C for 30 s; and a final extension at 72 °C for 10 min. The *T. drosophilae*-specific primers TRdroS (5′-TAATGATCAAATTTATAATTCTATTGTTACTT-3′) and TRdroR (5′-ATAAAATTGATCATACAAATAATCTTATTATATT-3′) amplify a 422-bp fragment, which was visualized by 2% agarose gel electrophoresis; samples showing the 422-bp band were scored as parasitized. Each run included a positive control (*T. drosophilae* adult DNA) and a negative control (water blank); primer specificity and assay sensitivity were validated as shown in Figures S1–S3.

From these data, we calculated for each replicate the overall parasitism rate (number of parasitized pupae/total hosts offered), as well as the numbers of emerged females, males, and total offspring. Sex ratio was calculated as the proportion of females among emerged adults.

### 2.4. Statistical Analysis

All statistical analyses were performed using R version 4.5.3 [33] (R Core Team, 2026) using the following packages: readxl (v1.4.5), dplyr (v1.1.4), and tidyr (v1.3.1) for data import and management; car (v3.1.3) for Type III likelihood-ratio χ^2^ tests; multcomp (v1.4.30) and multcompView (v0.1.10) for Tukey HSD tests and compact letter displays; survival (v3.8.6) and survminer (v0.5.2) for Kaplan–Meier survival analysis, log-rank tests, and pairwise comparisons with Benjamini–Hochberg adjustment; MASS (v7.3.65) for negative binomial GLMs; emmeans (v1.11.2.8) for estimated marginal means and Tukey-adjusted pairwise comparisons; and ggplot2 (v4.0.0) and scales (v1.4.0) for graphics. Longevity data were analyzed with a two-way ANOVA followed by Tukey’s HSD test. Results are presented as mean ± SE, with different letters indicating significant differences (*p* < 0.05). Survival data were analyzed using Kaplan–Meier survival curves and the log-rank test; pairwise comparisons among treatments were adjusted with the Benjamini–Hochberg method. Parasitism rate was analyzed using a generalized linear model (GLM) with a binomial distribution and logit link; overdispersion was assessed and, if present, a quasi-binomial correction was applied. Emergence counts (total, female, and male wasps) were analyzed with negative binomial GLMs (with a Poisson fallback if the negative binomial model failed to converge), and sex ratio (female proportion) with a binomial GLM on the counts of females and males. Models initially included treatment, log-transformed host density, and their interaction. When the interaction was non-significant, the interaction term was removed and results are reported from main-effect models with host density retained as a covariate; because diet effects were consistent across host densities, density-specific breakdowns are not presented for these variables. Significance of fixed effects in GLMs was evaluated by Type III likelihood-ratio χ^2^ tests, and pairwise comparisons among treatments were conducted using Tukey-adjusted contrasts on estimated marginal means. Post-hoc power analyses (α = 0.05) indicated that all significant diet effects were detected with power ≥ 0.89.

## 3. Results

### 3.1. Adult Parasitoid Longevity

**Figure 1:**
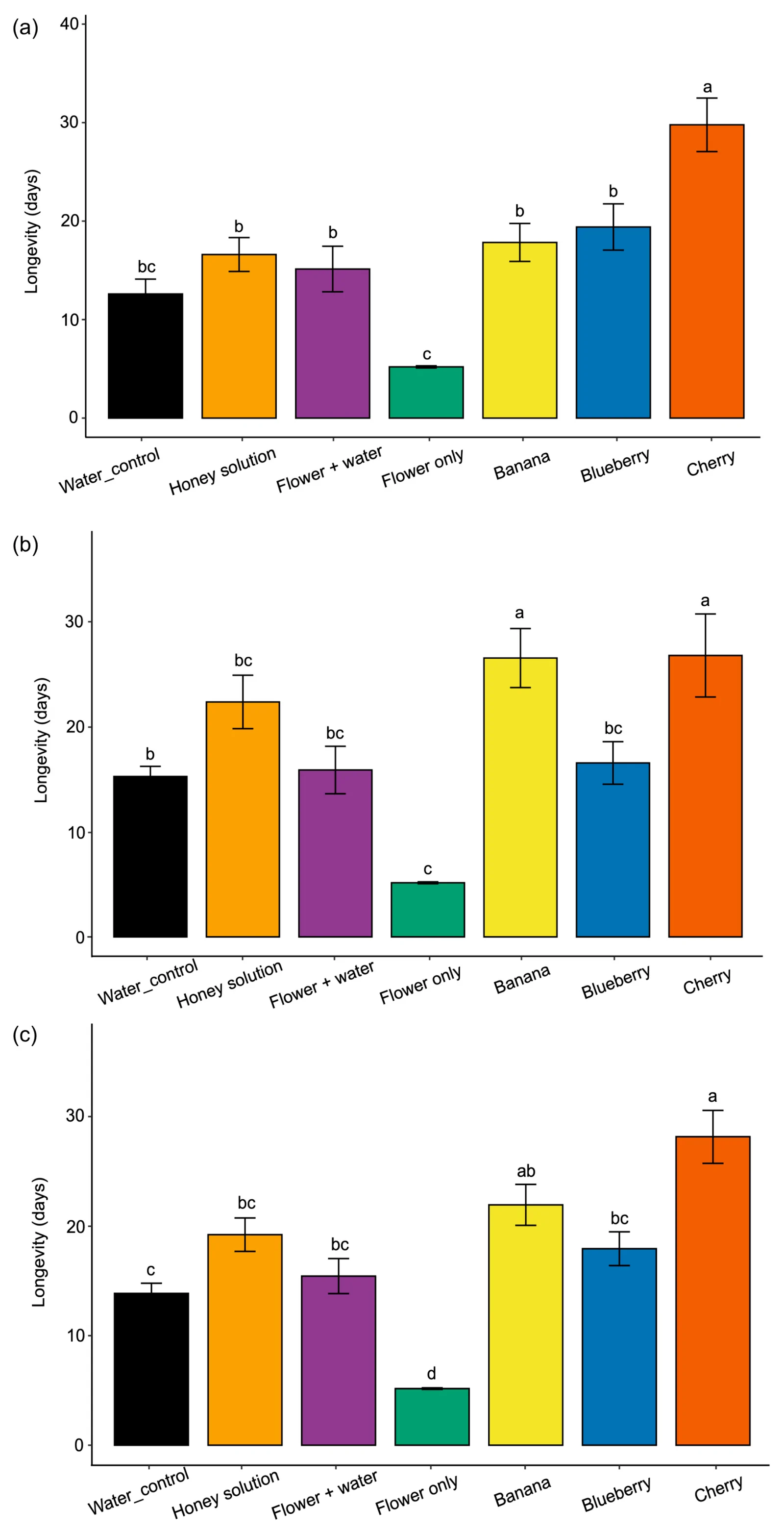
Longevity of parasitoid wasps fed on different food sources. (**a**) Females. (**b**) Males. (**c**) Overall (females and males combined). Bars represent mean ± SE. Different lowercase letters above bars indicate significant differences among treatments (Tukey’s HSD, *p* < 0.05). Treatments: Water_control, 10% Honey solution, Water + Flower, Flower only, Banana, Blueberry, and Cherry.

Adult longevity of the parasitoid wasps was evaluated across seven dietary treatments: Water control, 10% Honey solution, Water + Flower, Flower only, Banana, Blueberry, and Cherry. A total of 200 individuals (104 females, 96 males) were included in the analysis. The overall mean ± SE longevity was highest in the Cherry treatment (28.2 ± 2.40 days) and lowest in the Flower treatment (5.2 ± 0.07 days). Among females, mean longevity ranged from 5.2 ± 0.11 days (Flower) to 29.8 ± 2.72 days (Cherry). Among males, mean longevity ranged from 5.1 ± 0.09 days (Flower) to 26.7 ± 3.94 days (Cherry) (Figure 1).

A two-way ANOVA revealed a significant effect of treatment (*F*_6,186_ = 19.43, *p* < 0.001) and gender (*F*_1,186_ = 4.13, *p* = 0.044) on overall longevity, with no significant interaction (*F*_6,186_ = 1.78, *p* = 0.106). The Flower treatment had the shortest longevity (5.17 ± 0.07 days), significantly lower than all other treatments. At the opposite end, the Cherry treatment had the highest longevity (28.2 ± 2.40 days), significantly higher than Water control (13.9 ± 0.93 days), but not significantly different from Banana (22.0 ± 1.88 days), Honey solution (19.2 ± 1.53 days), Blueberry (18.0 ± 1.54 days), and Water + Flower (15.4 ± 1.60 days) treatments. No other pairwise comparisons were statistically significant (Figure 1c).

For female wasps, longevity also differed significantly among treatments (*F*_6,97_ = 11.47, *p* < 0.001). Cherry-fed wasps lived significantly longer (29.8 ± 2.72 days) than those fed any other diet. In contrast, flower-fed wasps had the shortest longevity (5.2 ± 0.11 days); despite this numerical difference, their longevity did not differ significantly from that of the water control (12.6 ± 1.51 days) (Tukey’s HSD, *p* = 0.1004), owing to the comparatively high variance of the control group. Intermediate longevity was recorded for wasps fed banana (17.8 ± 1.93 days), blueberry (19.4 ± 2.35 days), honey solution (16.6 ± 1.72 days), and water + flower (15.1 ± 2.31 days); these four values did not differ significantly from one another or from the control, but were significantly lower than that of cherry-fed wasps and significantly higher than that of flower-fed wasps (Tukey’s HSD test, *p* < 0.05) (Figure 1a).

For male wasps, longevity also varied significantly across treatments (*F*_6,89_ = 9.84, *p* < 0.001). Flower resulted in the shortest lifespan (5.13 ± 0.09 days), which was significantly lower than that of all other treatments. In contrast, Cherry (26.7 ± 3.94 days) and Banana (26.5 ± 2.80 days) produced the longest longevities; these two treatments were not significantly different from each other but were significantly longer-lived than the water control (15.2 ± 0.97 days). The remaining treatments—Honey solution (22.3 ± 2.53 days), Blueberry (16.5 ± 2.02 days), and Water + Flower (15.8 ± 2.25 days)—exhibited intermediate longevity and did not differ significantly from one another or from the water control (Figure 1b).

### 3.2. Adult Parasitoid Survival Rate

Survival analysis revealed significant differences in parasitoid wasp survival among dietary treatments (Log-rank test: χ^2^ = 186.39, df = 6, *p* < 0.0001). Compared to Water Control, several treatments significantly affected wasp survival.

**Figure 2:**
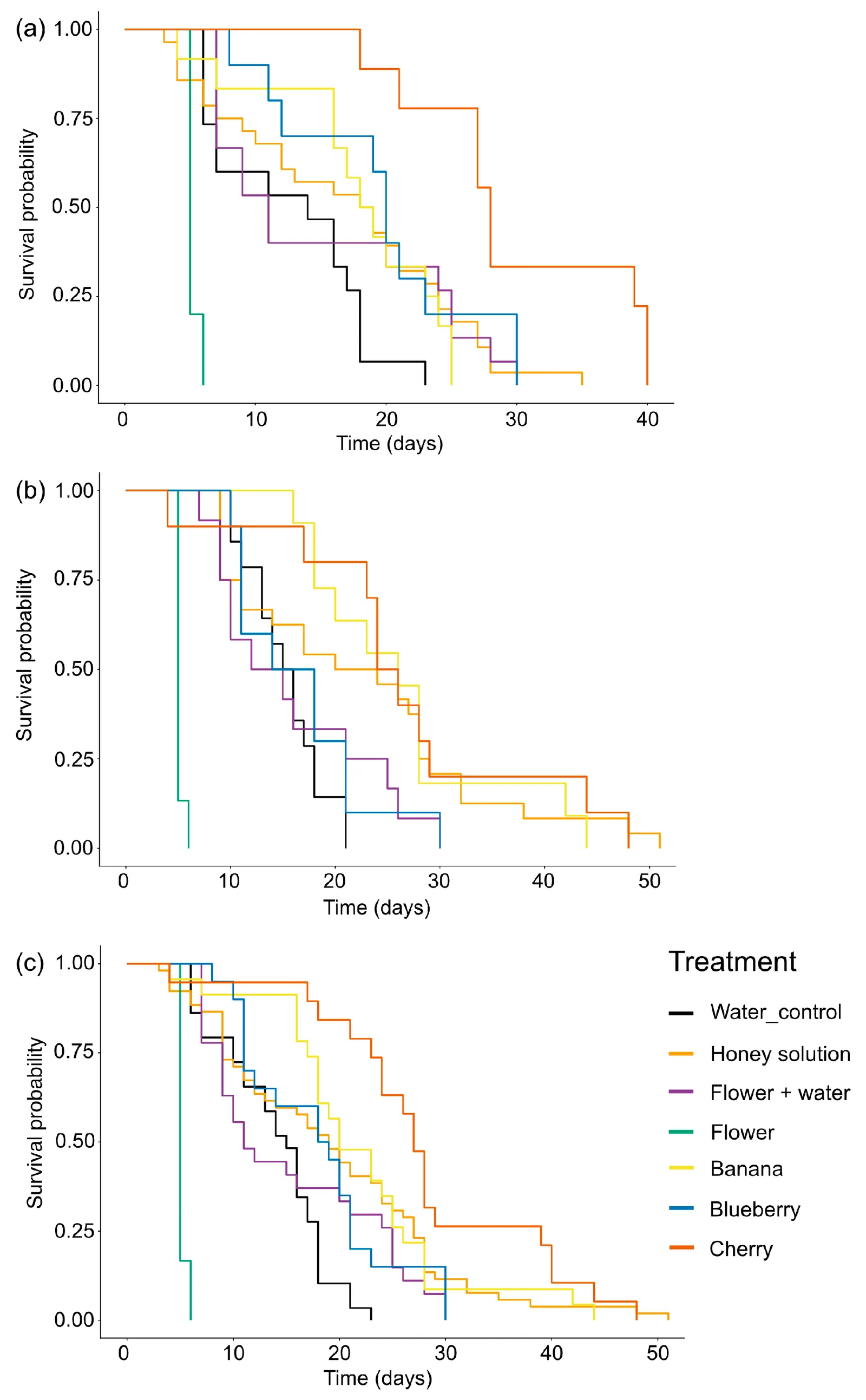
Survival curves of parasitoid wasps under different dietary treatments, analyzed by the Kaplan–Meier method. Statistically significant differences between treatment groups were determined by the log-rank test with pairwise post-hoc comparisons (*p* < 0.05). (**a**) Female survival (*n* = 104). (**b**) Male survival (*n* = 96). (**c**) Overall survival (*n* = 200). Treatments: Water_control, 10% honey solution, Water + Flower, Flower only, Banana, Blueberry, and Cherry.

Overall survival analysis (*n* = 200): Cherry and Banana treatments resulted in significantly higher survival rates compared to Water control (*p* < 0.001 for both comparisons), with hazard ratios of 0.074 (95% CI: 0.026–0.210) and 0.232 (95% CI: 0.117–0.459), respectively. Blueberry (HR = 0.446, 95% CI: 0.239–0.834, *p* = 0.0115) and 10% Honey solution (HR = 0.404, 95% CI: 0.239–0.685, *p* = 0.0008) also significantly improved survival compared to control. Water + Flower showed no statistically significant difference from control (HR = 0.603, 95% CI: 0.331–1.098, *p* = 0.0981). In contrast, Flower only treatment significantly reduced survival compared to control (HR = 31.992, 95% CI: 10.331–99.067, *p* < 0.0001) (Figure 2c).

Female-specific analysis (*n* = 104): Similar patterns were observed in female wasps (χ^2^ = 86.3, df = 6, *p* < 0.0001). Cherry showed the most pronounced survival benefit (HR = 0.064, 95% CI: 0.014–0.300, *p* = 0.0005), followed by Blueberry (HR = 0.305, 95% CI: 0.122–0.764, *p* = 0.0112), Banana (HR = 0.348, 95% CI: 0.146–0.830, *p* = 0.0173), and 10% Honey solution (HR = 0.438, 95% CI: 0.216–0.887, *p* = 0.0218). Water + Flower did not significantly differ from control (HR = 0.500, 95% CI: 0.219–1.141, *p* = 0.0999), while Flower only again significantly reduced survival (HR = 14.930, 95% CI: 4.511–49.413, *p* < 0.0001) (Figure 2a).

Male-specific analysis (*n* = 96): Male wasps also showed significant survival differences among treatments (χ^2^ = 111.87, df = 6, *p* < 0.0001). Banana (HR = 0.139, 95% CI: 0.044–0.439, *p* = 0.0008) and Cherry (HR = 0.085, 95% CI: 0.018–0.397, *p* = 0.0017) provided the greatest survival benefits, followed by 10% Honey solution (HR = 0.388, 95% CI: 0.174–0.862, *p* = 0.0200). Neither Blueberry (HR = 0.681, 95% CI: 0.289–1.607, *p* = 0.3805) nor Water + Flower (HR = 0.736, 95% CI: 0.310–1.751, *p* = 0.4883) significantly differed from control. Flower only treatment again significantly reduced male survival (HR = 3.100, 95% CI: 1.496–6.422, *p* < 0.0001) (Figure 2b).

### 3.3. Parasitism Rate

Overall, parasitism rates (mean ± SE) were 5.9% ± 1.4% for water control, 6.6% ± 1.4% for 10% honey solution, 21.7% ± 3.9% for banana, 23.2% ± 2.8% for blueberry, and 21.4% ± 3.1% for cherry. The parasitism rate was significantly influenced by the interaction between diet treatment and host density (χ^2^ = 11.40, df = 4, *p* = 0.022), and the overall density effect was also significant (χ^2^ = 5.55, df = 1, *p* = 0.018), while the treatment main effect was not significant when the interaction was included (χ^2^ = 8.20, df = 4, *p* = 0.084). Because the interaction was significant, treatment effects were examined separately for each host density level.

**Figure 3:**
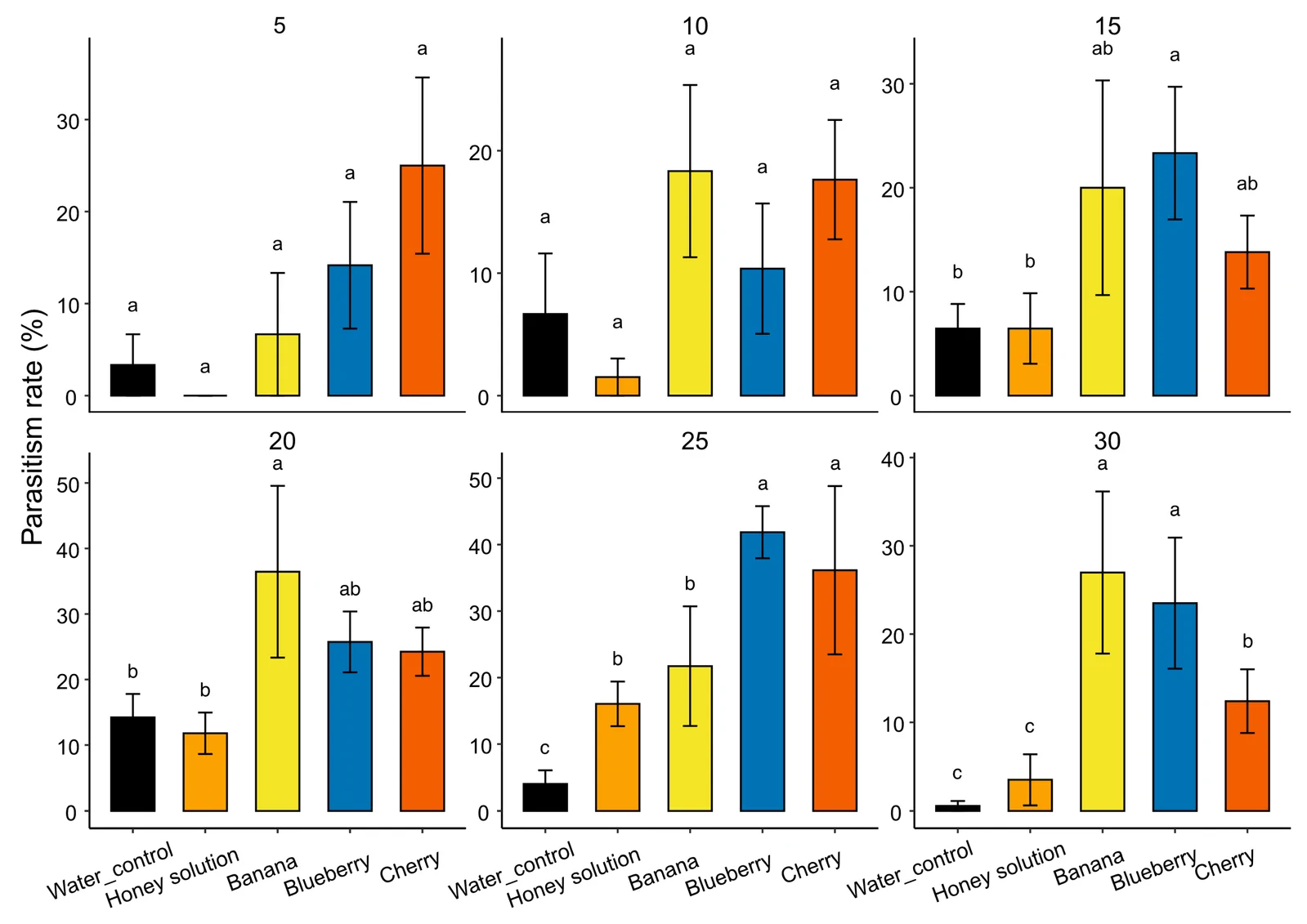
Parasitism rate (mean ± SE) of *Trichopria drosophilae* under different adult diet treatments (Water_control, 10% honey solution, Banana, Blueberry, and Cherry) across six host densities (5, 10, 15, 20, 25, 30). Within each density panel, different lowercase letters indicate significant differences among diet treatments (Tukey’s HSD, *p* < 0.05).

At densities of 5 and 10, no significant differences were detected among diet treatments, with parasitism rates ranging from 0.0% ± 0.0% (10% honey) to 25.0% ± 9.6% (cherry) at density 5, and from 1.5% ± 1.5% (10% honey) to 18.3% ± 7.0% (banana) at density 10. At density 15, blueberry (23.3% ± 6.4%) had a significantly higher parasitism rate than water control (6.5% ± 2.4%) and 10% honey solution (6.5% ± 3.4%). At density 20, banana (36.5% ± 13.1%) was the only treatment that significantly exceeded water control (14.2% ± 3.6%) and 10% honey solution (11.8% ± 3.2%). At density 25, all three fruit treatments (banana: 21.7% ± 9.0%, blueberry: 41.9% ± 3.9%, cherry: 36.1% ± 12.7%) showed significantly higher parasitism than water control (4.0% ± 2.0%); blueberry and cherry also surpassed 10% honey solution (16.1% ± 3.4%) and banana (21.7% ± 9.0%). At density 30, banana (27.0% ± 9.2%), blueberry (23.5% ± 7.4%), and cherry (12.4% ± 3.6%) all exhibited significantly higher parasitism than water control (0.6% ± 0.6%) and 10% honey solution (3.5% ± 2.9%) (Figure 3).

**Figure 4:**
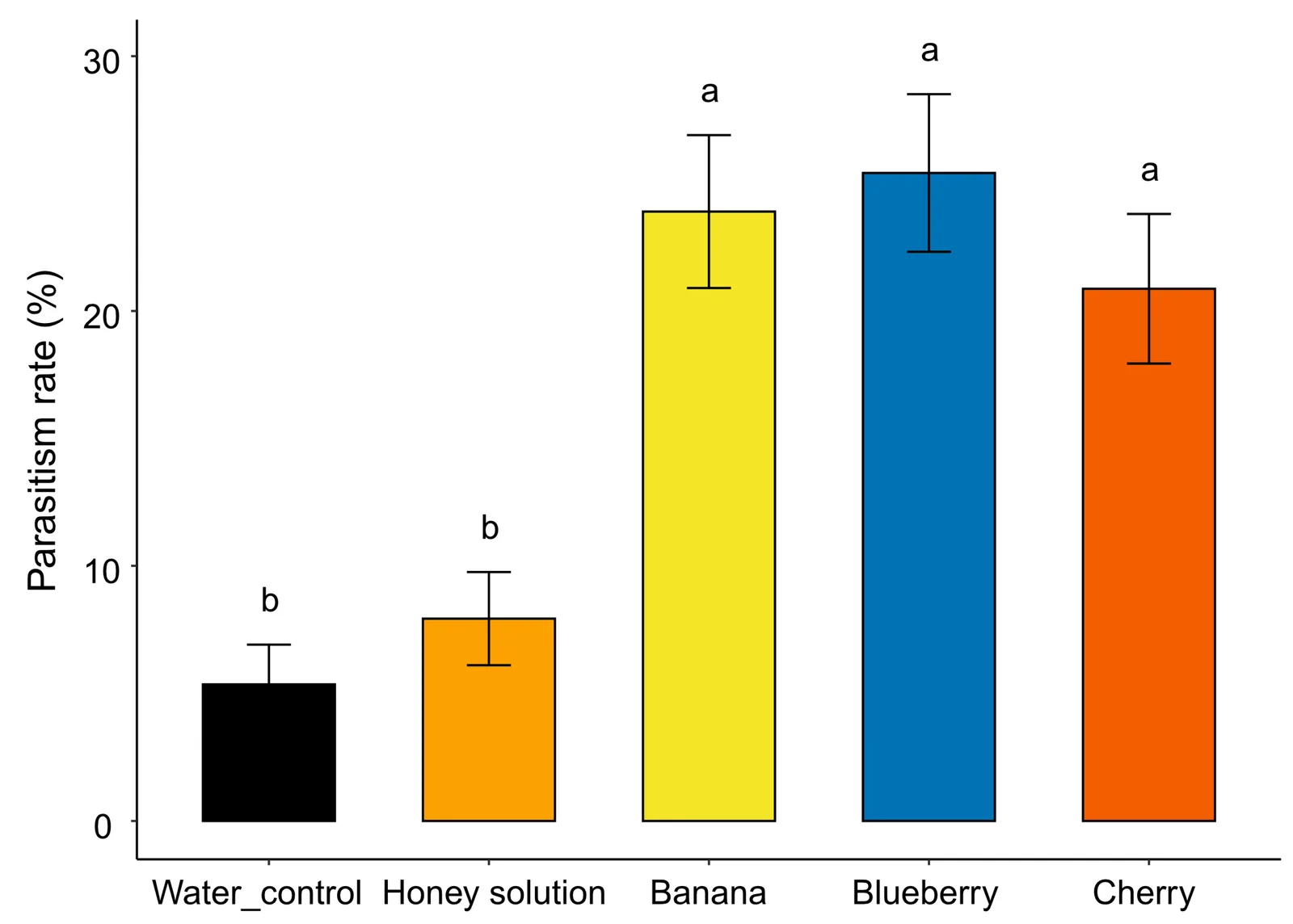
Parasitism rate (mean ± SE) of *Trichopria drosophilae* with different diet treatments: Water_control, 10% honey solution, Banana, Blueberry, and Cherry. Columns marked with different lowercase letters are significantly different (Tukey’s HSD, *p* < 0.05).

To provide an overall summary of treatment differences across densities, a reduced model without the interaction was also fitted. In this additive model, treatment was highly significant (χ^2^ = 59.61, df = 4, *p* < 0.001), with all three fruit treatments showing significantly higher parasitism than Water_control and 10% honey solution, whereas the density effect was only marginally significant (χ^2^ = 3.50, df = 1, *p* = 0.061) (Figure 4). Of the 1290 intact, uneclosed pupae tested by species-specific PCR, 312 yielded the 422-bp *T. drosophilae* fragment and were therefore scored as parasitized; these PCR-positive pupae were included in the parasitism counts reported above. Primer specificity (no amplification from *D. suzukii* or *D. melanogaster* adults or pupae) and assay sensitivity (detection limit of 1000 copies per reaction) are shown in Figures S1 and S2, and detection of parasitism 1 h after parasitization is shown in Figure S3.

### 3.4. Wasp Emergence and Sex Ratio

**Figure 5:**
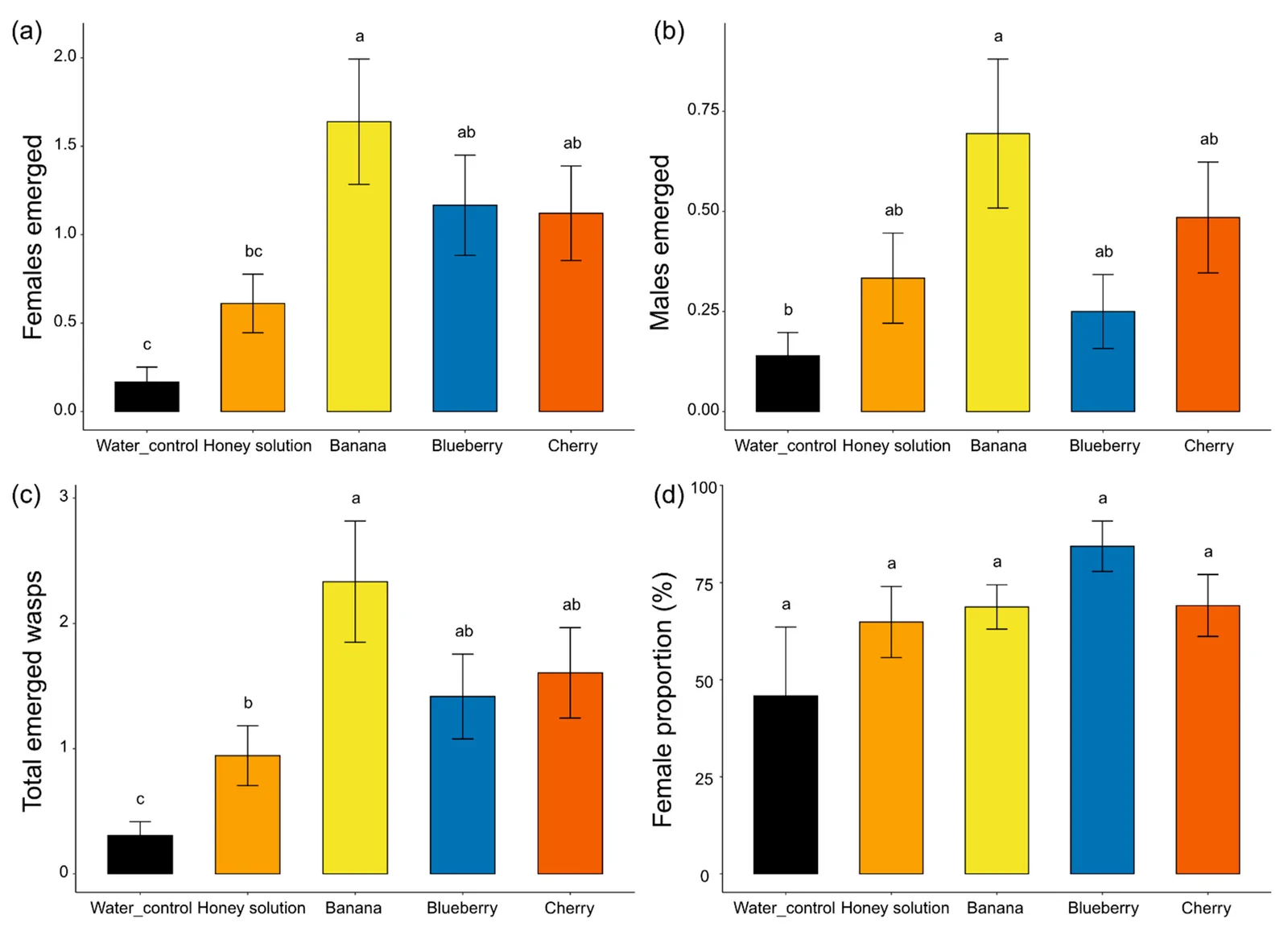
Effects of adult diet (water control, 10% honey, banana, blueberry, and cherry) on wasp emergence and offspring sex ratio of *Trichopria drosophilae*. Bars show observed means ± SE pooled across all host densities for display; statistical comparisons were based on GLMs with log-transformed host density included as a covariate. (**a**) Female emerged wasps, (**b**) male emerged wasps, (**c**) total emerged wasps, (**d**) sex ratio (proportion of females). Different lowercase letters indicate significant differences (Tukey-adjusted test, *p* < 0.05).

The total number of emerged wasps varied among treatments (χ^2^ = 33.89, df = 4, *p* < 0.001). Mean (±SE) total wasp counts were 0.31 ± 0.11 for Water control, 0.94 ± 0.24 for 10% honey solution, 2.33 ± 0.48 for Banana, 1.42 ± 0.34 for Blueberry, and 1.61 ± 0.36 for Cherry (*n* = 36 per treatment, except Cherry: *n* = 33). Water_control produced significantly fewer wasps than all other diets, including 10% honey solution (ratio = 0.32, *p* = 0.040), Banana (ratio = 0.13, *p* < 0.001), Blueberry (ratio = 0.25, *p* = 0.006), and Cherry (ratio = 0.23, *p* = 0.002). Emergence for 10% honey solution was significantly lower than Banana (ratio = 0.40, *p* = 0.019) but did not differ from Blueberry (*p* = 0.959) or Cherry (*p* = 0.823). No significant pairwise differences were found among Banana, Blueberry, and Cherry (all *p* ≥ 0.115) (Figure 5c).

Female emergence followed a similar pattern (χ^2^ = 36.19, df = 4, *p* < 0.001). Mean (±SE) female counts were 0.17 ± 0.08 for Water_control, 0.61 ± 0.17 for 10% honey solution, 1.64 ± 0.35 for Banana, 1.17 ± 0.28 for Blueberry, and 1.12 ± 0.27 for Cherry. Water_control had significantly lower female emergence than the fruit diets (Banana: ratio = 0.10, *p* < 0.001; Blueberry: ratio = 0.16, *p* = 0.001; Cherry: ratio = 0.17, *p* = 0.002), but not than 10% honey solution (ratio = 0.26, *p* = 0.054). Emergence for 10% honey solution was significantly lower than Banana (ratio = 0.37, *p* = 0.015) but did not differ from Blueberry (*p* = 0.563) or Cherry (*p* = 0.710). No significant differences were observed among the three fruit diets (all *p* ≥ 0.305) (Figure 5a).

Male emergence was also affected by treatment (χ^2^ = 14.86, df = 4, *p* = 0.005). Mean (±SE) male counts were 0.14 ± 0.06 for Water_control, 0.33 ± 0.11 for 10% honey solution, 0.69 ± 0.19 for Banana, 0.25 ± 0.09 for Blueberry, and 0.48 ± 0.14 for Cherry. The only significant pairwise difference was between Water_control and Banana (ratio = 0.20, *p* = 0.015); all other contrasts were non-significant (*p* ≥ 0.095) (Figure 5b).

Diet treatment did not significantly affect offspring sex ratio (proportion females) (χ^2^ = 5.22, df = 4, *p* = 0.266). Mean (±SE) female proportions were 0.46 ± 0.18 for Water_control, 0.65 ± 0.09 for 10% honey solution, 0.69 ± 0.06 for Banana, 0.84 ± 0.07 for Blueberry, and 0.69 ± 0.08 for Cherry. No pairwise comparison reached significance (all Tukey-adjusted *p* ≥ 0.330) (Figure 5d).

## 4. Discussion

This study provides a comprehensive evaluation of how different dietary resources influence the fitness of *T. drosophilae*, revealing significant and complex effects on its survival, longevity, and reproductive performance. As a promising biological control agent against *D. suzukii* and other drosophilid pests, strategies such as augmentative releases of *T. drosophilae* have shown potential for reducing pest populations in agroecosystems [5,34]. However, the efficacy of such biological control can be constrained by ecological factors, including the parasitoid’s limited access to host pupae located on the ground [35]. Therefore, enhancing key fitness parameters through nutritional supplementation is a crucial strategy for improving its field performance. Our results underscore the critical importance of selecting appropriate adult diets in both mass rearing and field-release programs to optimize the biological control services provided by this parasitoid.

The most pronounced finding was the stark contrast between the effects of fruit-based resources and floral resources on parasitoid fitness. Diets of cherry, banana, and blueberry pulps significantly enhanced adult longevity and survival compared to the water control, with cherry being particularly effective, extending the mean lifespan to over 28 days. This performance often matched or surpassed that supported by the standard 10% honey solution, consistent with previous findings that fruit pulp or juice can sustain parasitoid lifespans comparable to those achieved on honey [36,37]. In contrast, wasps that received only *O. violaceus* flowers lived for approximately 5 days on average, but the addition of water together with the flowers restored survival to a level statistically comparable to the water control. This pattern indicates water deprivation was an important (at least partial) cause of the short lifespan on flowers alone, likely combined with the absence of accessible sugars, and that water availability is critical for *T. drosophilae* survival when other food resources are unavailable. Notably, the Water + Flower treatment never significantly exceeded the Water control, indicating that the flowers provided no accessible nectar. Subsequent observation of *O. violaceus* flowers revealed structural barriers to nectar access for the parasitoid. The flowers have a broadly obovate shape [38] and a long corolla [26,39]. Nectar in such long-corolla flowers is held in narrow, deep tubes or spurs, typically accessible only to long-tongued insects like bumblebees and butterflies, but not to many natural enemies with shorter mouthparts [40]. Although *O. violaceus* flowers do produce nectar, it is effectively inaccessible to short-mouthed parasitoids such as *T. drosophilae*; accordingly, the ~5-day lifespan on flowers alone most plausibly reflects combined sugar and water deprivation rather than any toxic effect of the flower, because adding water together with the flowers restored survival to control levels. These observations together explain the nutritional inadequacy of this common orchard cover crop for *T. drosophilae* in early spring; direct quantification of nectar access (e.g., using dye-labelled nectar) was not performed and represents a useful avenue for future work. It is noteworthy, however, that other plant species flowering in early spring (e.g., *Brassica campestris*, *Diplotaxis tenuifolia*, *Lobularia maritima*, *Arachis hypogaea*, *Glycine max*, *F. esculentum*) could serve as alternative floral resources [40]. Critically, several of these, including *L. maritima*, *F. esculentum*, and *Vicia faba*, have been documented to enhance the pest control efficacy of various parasitoids [14,41,42], underscoring the importance of species-specific selection in conservation biological control programs.

The enhanced longevity and survival conferred by fruit resources directly translated into improved reproductive performance. Wasps fed cherry, blueberry, or banana pulp exhibited significantly higher overall parasitism rates and produced significantly more total offspring than water-fed wasps; among the fruit diets, banana additionally exceeded the honey treatment in both total and female offspring production. This aligns with the principle that improved adult nutrition fuels both somatic maintenance and reproductive investment. Although feeding behavior was not quantified directly, several lines of evidence indicate that the wasps consumed the fruit pulp. Fruit pulp was the sole nutrient source available in the fruit treatments, and fruit-fed wasps lived substantially longer than water-fed controls—an outcome that is impossible without ingested nutrients—and converted the acquired resources into higher parasitism and offspring production. Thus, the diet itself operated as the experimental variable, and its effects on survival, behavior-relevant energy reserves, and reproductive output were captured by our measurements. Direct quantification of feeding (e.g., using dye- or isotope-labeled substrates) and attraction assays would further strengthen this interpretation and are planned for future work. Beyond the direct nutritional benefits, fruit-based diets may also enhance parasitism indirectly through host-mediated effects. For example, Girod et al. (2018) demonstrated that several Asian larval parasitoids of *D. suzukii* achieved higher parasitism rates when their hosts developed on fresh blueberry fruit compared to artificial diet, suggesting that host larvae feeding on fresh fruit may be more suitable or attractive for parasitoid development [43]. Our results thus raise the possibility that fruit pulp not only nourishes adult parasitoids directly, but could also enhance the quality of host larvae developing within those fruits, thereby synergistically boosting overall parasitism success.

Interestingly, the beneficial effect of fruit diets on parasitism rate was not constant across host densities; a significant treatment × density interaction indicated that the nutritional advantage became increasingly apparent as host density increased. At low densities (5 and 10 hosts), parasitism rates were uniformly low across all treatments, whereas at intermediate to high densities (15–30 hosts), specific fruit treatments parasitized significantly more hosts than the water control (blueberry at 15 hosts; banana at 20 hosts; all three fruit treatments at 25 and 30 hosts). This density-dependent pattern may be partially explained by the role of nutrition in enhancing host-searching efficiency. Lightle et al. (2010) reported that sugar-fed *Apanteles aristoteliae* females were significantly more attracted to host-related cues than to food cues in olfactometer tests, whereas starved wasps showed no such preference [44]. Similarly, the energy reserves obtained from fruit consumption in our study may allow *T. drosophilae* females to prioritize host location over food searching, thereby sustaining higher oviposition rates when hosts are abundant. In contrast, at low host densities, even nutritionally limited females may be capable of handling the small number of available hosts, thus minimizing the observable advantage of fruit feeding. A simplified additive model, which ignored the interaction, confirmed that all fruit-fed groups had significantly higher overall parasitism than the water and honey controls; however, this main-effect conclusion should be viewed as a descriptive trend across densities rather than a universal statement.

Throughout the assays, fruit was provided in excess of consumption and replaced daily, so the observed treatment differences reflect dietary quality rather than food quantity, depletion, or desiccation. Ripeness was standardized to fully ripe fruits because sugar profiles change with ripening (e.g., starch-to-sugar conversion in banana); residual variation in fruit composition may have contributed to within-treatment variance but did not obscure the clear differences among treatments. The three fruit pulps also differed markedly in composition, which may partly explain their distinct effects on parasitoid performance. Sweet cherry is rich in glucose (8.4 g/100 g) and fructose (5.5 g/100 g) with negligible sucrose, and its dominant organic acid is malic acid (0.76 g/100 g) [45,46], thus combining a high content of readily assimilated monosaccharides with mild acidity and adequate moisture (82 g/100 g)—a profile consistent with its superior effect on adult longevity. Banana pulp had the highest total sugar content (15.8 g/100 g, including substantial sucrose) together with starch (3.7 g/100 g, converted to sugars during ripening) and the lowest water content (75 g/100 g) [45,47]; this energy-dense, soft substrate may sustain the highest offspring production, although it ferments and spoils readily. In contrast, blueberry pulp contained the lowest total sugars (9.4 g/100 g, almost exclusively glucose and fructose) and the highest water content (84 g/100 g), and its acidity is dominated by citric and quinic acids [48], yielding a more acidic substrate that may reduce palatability or feeding. These compositional differences are consistent with the overall pattern we observed: cherry offered the best balance of sugars, acidity, and moisture for adult survival, banana provided the greatest energy density and supported the highest offspring emergence, and the three fruits performed comparably in the parasitism assay. Volatile compounds may additionally modulate attraction and feeding, but were not measured here and warrant future investigation.

Beyond informing field-based conservation strategies, our findings offer direct insights for improving the mass rearing of *T. drosophilae* in laboratory settings. The identification of highly effective and readily available dietary supplements, such as cherry pulp, can optimize adult longevity and reproductive output in mass-rearing systems for augmentative release programs. However, factory-scale application has not yet been demonstrated and would require careful evaluation of cost, shelf life, and hygiene, because fresh fruit pulp ferments and spoils rapidly and could support microbial contamination or other unwanted organisms. From an applied perspective, our results highlight promising, practical avenues for improving *T. drosophilae*-based biocontrol, but they rest solely on laboratory data obtained with a factitious host. The sustained positive effects of fruit pulps suggest that damaged or overripe cherries, blueberries, or bananas left in orchards may provide a valuable nutritional subsidy for parasitoids; however, such fruits may also attract additional pests (e.g., drosophilids, vinegar flies, and ants) and promote decay, so the net benefit for biological control should be weighed against these risks and validated under field conditions. This could enhance parasitoid persistence and pest suppression, potentially more effectively than artificial sugar feeders. While prior research indicates that microbial volatiles from fly-infested fruits can aid parasitoids in host location [49], our study provides a critical complement to this mechanism. We demonstrate that the nutritional resources from fruits can directly enhance parasitoid fitness, thereby synergistically improving overall biocontrol potential. Conversely, the strong negative effect of *O. violaceus* flowers alone necessitates a careful evaluation of floral resources within conservation biological control strategies. Future work should focus on the field and semi-field validation of these nutritional resources, quantification of contamination risks under mass-rearing conditions, analysis of the specific nutritional and phytochemical components (e.g., sugars, amino acids, secondary metabolites) responsible for the observed effects, and translation of the key findings to *D. suzukii* pupae, the primary target host in the field. Ultimately, tailoring the nutritional environment to support key natural enemies like *T. drosophilae* is a crucial step toward developing resilient and effective biological control systems.

In conclusion, this study provides a comprehensive evaluation of how different dietary resources influence the fitness of *T. drosophilae*, revealing significant and complex effects on survival, longevity, and reproductive performance. The results underscore the critical importance of selecting appropriate nutritional supplements in both laboratory rearing and, pending field validation, in field-release programs.

## Data Availability

Data supporting the results of this study can be obtained from the corresponding author upon request.

## Supplementary Materials

The following supporting information can be downloaded at: https://www.mdpi.com/article/10.3390/insects17090976/s1, Figure S1: Specificity of the TRdroS/TRdroR primers. Lane M, DL2000 DNA marker; lanes 1–3, *Drosophila suzukii* adults; lanes 4–6, *D. suzukii* pupae; lanes 7–9, *D. melanogaster* adults; lanes 10–12, *D. melanogaster* pupae; lanes 13–16, *Trichopria drosophilae* adults; lane 17, water blank. The 422-bp band appears only in *T. drosophilae*; Figure S2: Sensitivity of the TRdroS/TRdroR assay. Lane M, DL2000 DNA marker; lanes 1–12, ten-fold serial dilutions of *T. drosophilae* DNA from 1 × 10^10^ to 10 copies per reaction; lanes 13–14, water blanks. The 422-bp band is detectable down to 1000 copies; Figure S3: Detection of *T. drosophilae* DNA in parasitized host pupae. Lane M, DL2000 DNA marker; lane 1, water blank; lane 2, *T. drosophilae* adult; lanes 3–6, *D. melanogaster* pupae 1 h after exposure to *T. drosophilae*; lanes 7–10, *D. suzukii* pupae 1 h after exposure.
