# Supplementary material for: Fruit Pulps Enhance Longevity and Parasitism of Trichopria drosophilae, a Pupal Parasitoid of Drosophilid Flies
Source: Insects. 2026 Sep 21;17(9):976. doi: 10.3390/insects17090976 (PMC13607223; doi:10.3390/insects17090976)

Supplementary materials

Figure S1. Specificity of the TRdroS/TRdroR primers. Lane M, DL2000 DNA marker; lanes 1–3, *Drosophila suzukii* adults; lanes 4–6, *D. suzukii* pupae; lanes 7–9, *D. melanogaster* adults; lanes 10–12, *D. melanogaster* pupae; lanes 13–16, *Trichopria drosophilae* adults; lane 17, water blank. The 422-bp band appears only in *T. drosophilae*.

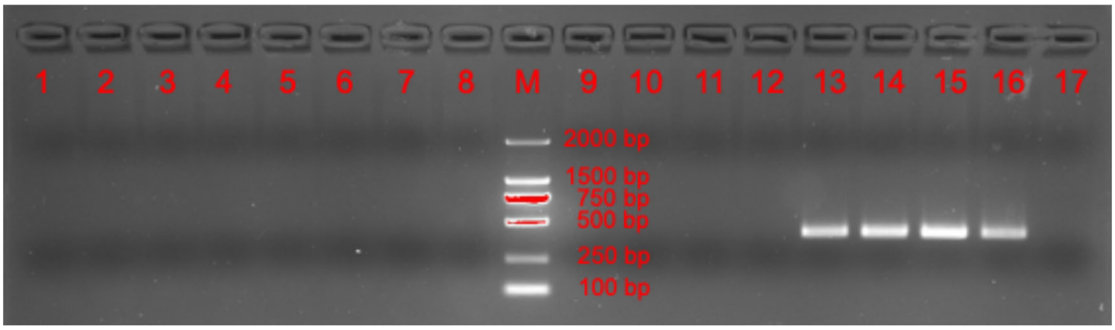

Figure S2. Sensitivity of the TRdroS/TRdroR assay. Lane M, DL2000 DNA marker; lanes 1–12, ten-fold serial dilutions of *T. drosophilae* DNA from 1 × 10^{10} to 10 copies per reaction; lanes 13–14, water blanks. The 422-bp band is detectable down to 1000 copies.

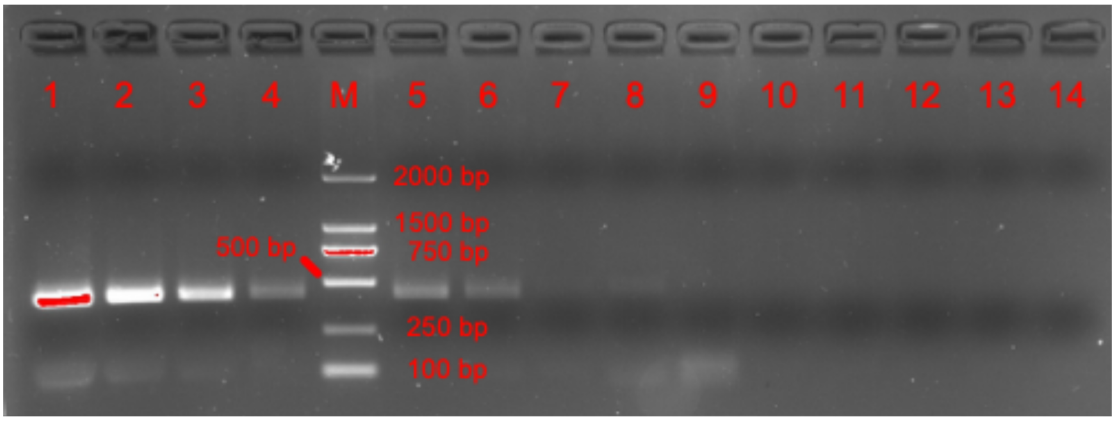

Figure S3. Detection of *T. drosophilae* DNA in parasitized host pupae. Lane M, DL2000 DNA marker; lane 1, water blank; lane 2, *T. drosophilae* adult; lanes 3–6, *D. melanogaster* pupae 1 h after exposure to *T. drosophilae*; lanes 7–10, *D. suzukii* pupae 1 h after exposure.

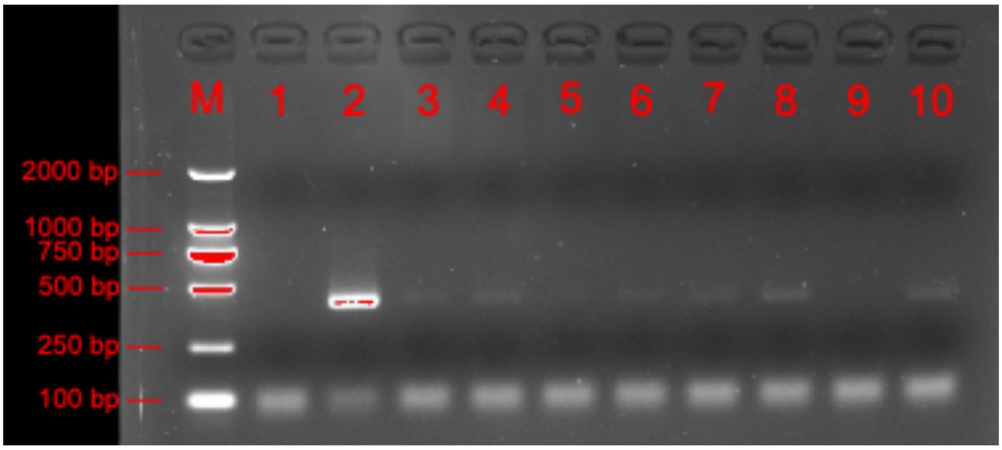

Supplement: Supplementary file 1 [file insects-17-00976-s001.zip › insects-4530313-supplementary.pdf]
